# Hierarchy and Speed of Loss in Physical Functioning: A Comparison Across Older U.S. and English Men and Women

**DOI:** 10.1093/gerona/glw209

**Published:** 2016-10-17

**Authors:** Rebecca Bendayan, Rachel Cooper, Elizabeth G. Wloch, Scott M. Hofer, Andrea M. Piccinin, Graciela Muniz-Terrera

**Affiliations:** 1 MRC Unit for Lifelong Health and Ageing at UCL, London, UK.; 2 Department of Psychology, University of Victoria, British Columbia, Canada.; 3 Centre for Dementia Prevention, University of Edinburgh, UK.

**Keywords:** Activities of daily living, Aging, Decline, Mobility

## Abstract

**Background:**

We aimed to identify the hierarchy of rates of decline in 16 physical functioning measures in U.S. and English samples, using a systematic and integrative coordinated data analysis approach.

**Methods:**

The U.S. sample consisted of 13,612 Health and Retirement Study participants, and the English sample consisted of 5,301 English Longitudinal Study of Ageing participants. Functional loss was ascertained using self-reported difficulties performing 6 activities of daily living and 10 mobility tasks. The variables were standardized, rates of decline were computed, and mean rates of decline were ranked. Mann–Whitney *U* tests were performed to compare rates of decline between studies.

**Results:**

In both studies, the rates of decline followed a similar pattern; difficulty with eating was the activity that showed the slowest decline and climbing several flights of stairs and stooping, kneeling, or crouching the fastest declines. There were statistical differences in the speed of decline in all 16 measures between countries. American women had steeper declines in 10 of the measures than English women. Similar differences were found between American and English men.

**Conclusions:**

Reporting difficulties climbing several flights of stairs without resting, and stooping, kneeling, or crouching are the first indicators of functional loss reported in both populations.

Physical functioning is commonly included in definitions of healthy aging (1,2). A range of complementary self-reported and performance-based measures are widely used to capture different aspects of physical functioning including questions about mobility and the ability to perform activities of daily living (ADL) such as having difficulties walking across a room, dressing, bathing and showering, eating, and getting in and out of bed or using the toilet (3).

First reported difficulties performing physical tasks of everyday living usually indicate an early stage of the disablement process (4,5) and have been associated with morbidity and mortality (6–8). Cross-sectional research has provided consistent evidence of a general pattern of loss in different functional tasks, which appears to follow a hierarchical order (5,9,10). For example, a study described consistent patterns of loss when single activities were grouped according to underlying impairments in five European countries (9). They found that older adults first lose the ability to perform activities that required balance, agility, and strength (eg, doing heavy housework, walking at least 400 m, or using stairs) and that activities that only required the use of upper extremities (eg, eating or washing arms and face) were the last to be lost. Similar patterns have been reported by other authors in different study populations (5,10,11).

Identifying activities with which people first report difficulty and the subsequent pattern of progression could be key to developing effective interventions that prevent or delay functional decline and to identify subgroups of individuals who may benefit from targeted intervention. Although existing reports provide consistent evidence of general patterns of loss based on prevalence, a longitudinal approach is needed to improve our understanding of the early stages of functional decline and in particular to help clarify early indicators of this process and determine how consistent these are across countries. Some studies have examined whether the hierarchy of functional loss is also found in longitudinal analyses (12–16). As difficulty performing ADLs and mobility-related tasks are usually captured using categorical scales (analyzed as binary variables), and data are often not captured on the timing of onset of difficulties, estimating rates of change over time can constitute a challenge. Two main approaches have been adopted to overcome this. First, some studies have examined change by comparing the total number of difficulties reported at different time points (4,6). Although this approach provides an estimate of change in overall physical functioning, individual information about the rate of change in each task cannot be extracted. Second, some studies have compared the median age at onset of disability for each variable (13,14,16). Although this approach provides longitudinal information for each variable, rates of change can still not be estimated. Within this context, the pattern of hierarchy of loss suggested in previous cross-sectional research has not been yet explored in terms of rate of change over time and further research is therefore needed.

Nevertheless, the above-mentioned studies that have tried to examine longitudinal change provided results that are in line with the findings from cross-sectional studies: measures of mobility (eg, climbing stairs, squatting, or walking half a mile) are the first set of activities people report difficulty with, whereas basic ADLs (eg, eating or toileting) appear to be the last. However, differences in the orderings are found across different studies. Inconsistencies in results could be due to the different methodological approaches adopted, the different tasks considered, or cultural differences associated with the different study settings. First, as it has been said, some studies examined change by comparing the number of difficulties at different time points (4,6), whereas other studies compared the median age of onset of disability (13,14,16). Second, some studies only focused on ADLs (12,13), whereas other studies also included mobility measures (14,16). Third, although some studies used English populations (13,14), others used American samples (6,12,15) or samples from other countries (4,16).

The importance of examining functional decline in older adults from an international perspective has been discussed thoroughly (17,18). Therefore, to facilitate a fair comparison of loss in physical functioning in different populations, the present study adopts a systematic and coordinated approach to examine the decline in self-reported physical functioning in the Health and Retirement Study (HRS), a nationally representative sample of older American adults, and the English Longitudinal Study of Ageing (ELSA), a nationally representative sample of older English adults. More specifically, we aim to identify the hierarchy of rates of decline in 6 basic ADLs and 10 mobility measures over 8 years, considering potential sex and country differences.

## Methods

### Study Populations

The U.S. sample consisted of a subsample of 13,612 respondents (41.25% men) of the HRS. The English sample consisted of a subsample of 5,301 respondents (43.40% men) of the ELSA. HRS and ELSA are sister studies, and both are biannual, longitudinal, and nationally representative surveys that focus on adults aged 50 and over. Data from two waves of each study have been used in these analyses: Wave 4 (1998) and Wave 8 (2006) of HRS and Wave 1 (2002) and Wave 5 (2010) of ELSA. These specific waves were selected to allow for the 10-year difference between the first wave of HRS and the first wave of ELSA and to facilitate the comparison of both samples in terms of age, period, and cohort. More detail on these studies can be found elsewhere (19,20). From now on, the first wave considered in each study in our analyses will be labeled as baseline and the fourth as (8-year) follow-up. Relevant descriptive statistics are provided in Table 1.

**Table 1.** Sample Descriptive Statistics for American (HRS) and English (ELSA) Men and Women

	Women		Men	
	HRS (*N* = 7,996)	ELSA (*N* = 3,000)	HRS (*N* = 5,616)	ELSA (*N* = 2,301)
Baseline age	64.17 (9.14)	61.41 (8.38)	63.72 (8.22)	60.81 (7.88)
Education				
No formal education	0.8%	0.6%	0.9%	0.3%
9 y or less	15.2%	17.3%	16.4%	17.7%
10–13 y	56.8%	70.1%	45.4%	65.2%
14 y or more	28.1%	12%	37.2%	16.8%
SRH at baseline				
Excellent/very good/good	74.2%	76.7%	77.1%	78.8%
Fair/poor	25,7%	23.3%	22.9%	21.2%
SRH at follow-up				
Excellent/very good/good	67.4%	69.3%	69.9%	71.5%
Fair/poor	32.6%	23.3%	30.1%	16.8%
Reported difficulties at baseline				
EAT	1.6%	1.6%	1.1%	0.7%
TOILET	4.8%	2.9%	1.9%	2.1%
WALKROOM	4.4%	2.1%	2.4%	1.2%
BED	5.5%	5.9%	3.8%	4.1%
BATH	4.8%	10.2%	2.5%	6.2%
DRESS	6.8%	10.4%	6%	11.9%
DIME	5.5%	5%	4.2%	3%
WALK100	9.9%	8.7%	5.5%	7.5%
SIT	19.9%	14.5%	14.1%	12.1%
ARMS	14.4%	11.1%	11.4%	7.2%
CLIMB1	14.8%	12.5%	7.1%	7.6%
PUSH	25.6%	17.9%	11.9%	8.4%
CHAIR	37.1%	28%	27.5%	20.4%
LIFT	23.5%	28.9%	9.4%	11.6%
STOOP	41.3%	36.4%	30.5%	27.7%
CLIMB SEVERAL	44.6%	39.6%	25.5%	23.2%

*Note:* SRH = self-rated health.

### Measures

In both studies, a standard set of questions on difficulty performing a range of ADLs (21) were asked at each wave. Individuals were asked if they had difficulties with the following activities: walking across a room (WALK ROOM), dressing (DRESS), bathing and showering (BATH), eating (EAT), getting in and out of bed (BED), and using the toilet (TOILET). Difficulty performing mobility tasks were also assessed in both studies at each wave, these were walking 100 yards/a block (WALK100), sitting for about 2 hours (SIT), getting up from a chair after sitting for long periods (CHAIR), climbing several flights of stairs without resting (CLIMB SEVERAL), climbing one flight of stairs without resting (CLIMB1), lifting or carrying weights over 10 lbs (LIFT), stooping, kneeling, or crouching (STOOP), reaching arms above shoulder level (ARMS), pushing or pulling large objects (PUSH), and picking up a 5p coin/dime from the table (DIME). Answers to each of these 16 questions were coded as 0 if no difficulties were reported or 1 if any difficulty was reported.

Self-rated health (SRH) was used to standardize the functioning measures, and it was assessed using an item that asked individuals to rate their health as excellent, very good, good, fair, or poor. A derived binary indicator grouped the ratings in two categories: excellent/very good/good versus fair/poor.

### Statistical Analyses

In order to examine change in the 16 variables considered and facilitate comparison of the changes within and between studies, the procedure proposed by Diehr and colleagues (22) was followed. This procedure consists of standardizing all the variables to a 100-point scale, using SRH as the standard followed by the calculation of the rates of change for each individual. Following the procedure of Diehr and colleagues (22), each variable was standardized by transforming them to the percent probability of being healthy (being healthy defined as reporting excellent/very good/good health at the next wave). That is, each original value was replaced with the percent of individuals at that value who had excellent, very good, or good SRH at the next wave. For example, for CHAIR, U.S. women with no difficulties at follow-up were allocated a score of 43.3 and those who had difficulties as 24.3 because 43.3% of U.S. women in the reference data set who reported not having difficulties with getting up from a chair reported that their health was excellent, very good, or good compared with only 24.3% of those that reported having difficulties with this task. As Diehr and colleagues (22) highlight, SRH is an appropriate measure against which to standardize ADL and mobility variables and the only requirement for this standardization is that SRH has to be monotonically related to the 16 variables considered. This requirement was met for all the measures in both samples. One of the main advantages of using this standardization procedure in our study is that the resulting standardized variables are on a scale that assumes that the change of a certain number of points has the same interpretation at every baseline level, and so rates of change can be computed from one measurement occasion to another.

Once the variables were standardized, rates of decline were computed for each person and for each variable and then the mean rates of decline were ranked within each study. In order to compare the rates of decline between countries, Mann–Whitney *U* tests for independent samples were performed and effect sizes were computed. Sensitivity analyses were performed to compare the order of the rates of change in the 16 measures in those individuals whose slopes were higher than the median (ie, faster decliners) with those individuals whose slopes were lower than the median (ie, slower decliners). We also reran the main analyses on four age bands separately (50–65, 65–70, 70–75, 75+).

## Results

Within each study, there were statistically significant sex differences in all slopes (*p* < .05), except for DRESS in ELSA justifying sex stratification of models when comparing differences between the U.S. and English samples. Table 2 shows the ranking of decline over time for the 16 functional measures for women and men, in both samples. In general, the activities for which fewer respondents reported difficulties were the ADLs and picking up a dime from the table. Those for which more respondents reported having difficulties were stooping and climbing several flights of stairs. Moreover, the slowest rate of decline was found for eating and the fastest rate of decline for climbing several flights of stairs without resting and stooping, kneeling, or crouching. (See Supplementary Figure 1 that shows the rate of decline for men and women in both samples for selected measures.)

**Table 2.** Ranking of Decline in 6 Basic ADL and 10 Mobility Measures Between Baseline and 8-Year Follow-up Among American (HRS) and English (ELSA) Women and Men

	Women		Men	
Rate of Decline	HRS	ELSA	HRS	ELSA
SLOWEST	EAT	EAT	EAT	EAT
	BED	TOILET	TOILET	WALK ROOM
	TOILET	WALK ROOM	BED	TOILET
	WALK ROOM	BED	BATH	BED
	BATH	DIME	DIME	DIME
	DIME	BATH	WALK ROOM	BATH
	DRESS	WALK100	DRESS	ARMS
	ARMS	DRESS	CLIMB1	WALK100
	CLIMB1	SIT	LIFT	CLIMB1
	SIT	ARMS	ARMS	PUSH
	LIFT	CLIMB1	PUSH	SIT
	PUSH	PUSH	SIT	LIFT
	WALK100	CHAIR	CHAIR	DRESS
	CHAIR	LIFT	WALK 100	CHAIR
	CLIMB SEVERAL	STOOP	CLIMB SEVERAL	CLIMB SEVERAL
FASTEST	STOOP	CLIMB SEVERAL	STOOP	STOOP

*Note:* ADL = activities of daily living; ELSA = English Longitudinal Study of Ageing; HRS = Health and Retirement Study. Eating (EAT), walking across a room (WALK ROOM), dressing (DRESS), bathing and showering (BATH), eating (EAT), getting in and out of bed (BED), using the toilet (TOILET), walking 100 yards/block (WALK100), sitting for about 2 hours (SIT), getting up from a chair after sitting for long periods (CHAIR), climbing several flights of stairs without resting (CLIMB SEVERAL), climbing one flight of stairs without resting (CLIM1), lifting or carrying weights over 10 lbs (LIFT), stooping, kneeling, or crouching (STOOP), reaching arms above shoulder level (ARMS), pushing or pulling large objects (PUSH), and picking up a 5p coin from the table (DIME).

In both men and women, our results indicate differences by country in rate of decline in each of the 16 measures considered (Table 3). Specifically, U.S. women had a steeper decline, compared to English women, in activities such as eating, using the toilet, walking across a room and getting in and out of bed, picking up a dime from the table, walking 100 yards/a block, sitting for about 2 hours, getting up from a chair after sitting for long periods, climbing one flight of stairs without resting, reaching arms above shoulder level, and pushing or pulling large objects. However, English women had a steeper decline, compared to U.S. women in the other six tasks. The same pattern of differences was found between American and English men, except for getting in and out of bed where English men had a steeper decline compared to U.S. men.

**Table 3.** Rates of Change in 6 Basic ADL and 10 Mobility Measures Between Baseline and 8-Year Follow-up Among American (HRS) and English (ELSA) Women and Men

	Women				Men			
	HRS Slope	ELSA Slope	*U*	*r*	HRS Slope	ELSA Slope	*U*	*r*
	*M* (*SD*)	*M* (*SD*)			*M* (*SD*)	*M* (*SD*)		
EAT	9.96 (12.23)	8.90 (9.36)	10,690,866*	.04	9.18 (10.64)	8.26 (7.30)	10,690,866*	.04
TOILET	13.63 (16.22)	10.35 (12.13)	724,470*	.80	9.76 (11.72)	9.47 (10.39)	724,470*	.91
WALK ROOM	13.84 (17.14)	10.33 (12.36)	748,416*	.86	11.39 (14.34)	8.89 (9.83)	748,416*	.92
BED	12.73 (15.87)	11.88 (14.87)	1,130,130*	.82	10.72 (13.59)	10.96 (13.83)	1,130,130*	.88
BATH	14.42 (17.47)	18.21 (20.01)	1,628,520*	.78	11.24 (14.25)	14.36 (17.95)	1,628,520*	.87
DRESS	15.23 (18.10)	19.44 (21.08)	1,769,674*	.74	15.74 (17.96)	21.37 (21.08)	1,769,674*	.76
DIME	14.99 (16.68)	14.07 (16.60)	1,531,281*	.79	13.36 (15.37)	11.98 (14.41)	546,700*	.86
WALK100	34.26 (18.53)	18.71 (21.32)	2,774,352*	.60	29.52 (21.75)	16.65 (20.03)	1,178,220*	.68
SIT	26.96 (19.40)	19.98 (19.89)	2,798,984*	.63	23.95 (19.79)	18.74 (19.78)	1,313,450*	.68
ARMS	23.73 (20.17)	20.05 (20.12)	2,858,643*	.64	21.28 (19.69)	16.39 (18.48)	1,048,704*	.74
CLIMB1	25.08 (21.68)	23.65 (21.90)	3,736,888*	.54	18.76 (20.85)	17.40 (20.02)	1,218,204*	.70
PUSH	33.01 (18.13)	28.40 (22.11)	4,708,732*	.42	21.45 (21.50)	17.92 (20.97)	1,329,750*	.68
CHAIR	39.40 (9.44)	34.21 (18.68)	6,723,648*	.30	37.31 (15.09)	28.92 (21.38)	2,635,576*	.45
LIFT	31.46 (19.62)	35.10 (19.34)	4,090,971*	.49	19.16 (20.87)	19.84 (21.63)	1,324,895*	.69
STOOP	40.38 (4.44)	40.70 (11.23)	9,453,507*	.08	39.36 (12.36)	36.38 (17.60)	3,760,786*	.29
CLIMB SEVERAL	40.25 (3.78)	41.11 (10.87)	8,848,372*	.05	37.09 (16.42)	34.58 (20.08)	3,304,226*	.31

*Note:* ADL = activities of daily living; ELSA = English Longitudinal Study of Ageing; HRS = Health and Retirement Study; *M* = mean; *r* = effect size; *SD* = standard deviation; *U* = Mann–Whitney tests. Women of HRS begin with 74.2 and in ELSA with 76.7; men of HRS begin with 77.1 and in ELSA with 78.8.

*p < .0001.

In sensitivity analyses, attrition and missing data patterns were examined. Overall attrition from baseline to follow-up was 1.3% for men and 0.9% for women in HRS and 6.9% for men and 7.0% for women in ELSA. Although there was higher attrition in ELSA than HRS, the percentage lost to follow-up due to mortality was similar in men and women in both studies (around 1%). Overall, those individuals who were still alive at follow-up had higher levels of education, reported better health status, and were less likely to report difficulties with ADL and mobility tasks at baseline compared to those that were not alive at follow-up. Additional sensitivity analyses were performed to compare the order of the rates of change in the 16 measures in those individuals whose slopes were higher than the median (ie, faster decliners) with those individuals whose slopes were lower than the median (ie, slower decliners). A similar pattern was found in slower and faster decliners, for U.S. and English men and women, and this pattern was consistent with the patterns found in men and women in HRS and ELSA. When considering different age groups separately (ie, 50–65, 65–70, 70–75, 75+), similar overall patterns were also found but the rates of decline were higher in the older groups compared to the younger ones. Furthermore, the average initial levels of health were slightly higher in the age-restricted cohorts than in the total sample. This could be associated with a survivor effect (6). Differences observed in the rates of decline in the English men aged 70–75 suggest that there might be some birth cohort differences.

## Discussion

The present study aimed to identify the hierarchy of rates of decline in 6 basic ADLs and 10 mobility measures over 8 years in older adults, considering potential sex and country differences.

In general, the results showed that a hierarchical pattern of rates of decline can be found in both samples; the six ADLs showed a slower rate of decline over time than the mobility tasks with eating consistently found to have the slowest decline and climbing several flights of stairs without resting, and stooping, kneeling, or crouching, the fastest declines. Although the other eight measures were not ranked in the same order across samples, in general, the rate of decline of the ADLs and picking up a dime from a table showed the slowest decline and some of the mobility measures that require the use of lower extremities showed the fastest. These results were consistent with previous research that has identified a hierarchical pattern of loss in physical functioning (5,9–16).

Differences in rates of decline were found between the U.S. and English samples. In general, English participants appeared to have higher levels of physical functioning and a slower rate of decline in most of the activities considered compared to their American counterparts. These results are consistent with previous research that found that English older adults are generally healthier than American older adults (23,24) and less disabled (25). However, for some activities, such as bathing and showering, dressing, lifting or carrying weights over 10 lbs, climbing several flights of stairs without resting, and stooping, kneeling, or crouching, English individuals appeared to decline faster than U.S. individuals. It should be noted that in both studies the ADL and mobility measures that had the highest prevalence of difficulty at baseline declined more quickly over follow-up.

One of the main advantages of the present study is that its design and statistical analysis facilitates comparability across samples and future replication studies. Replication studies are essential to build scientific knowledge, and recent publications have highlighted the need to promote systematic replication efforts (26,27), especially in longitudinal studies of aging (28). However, such studies are challenging as there are a number of sources of variability that can confound the results. The variety of measures used to assess the same constructs in different studies constitutes one of the greatest challenges. The present study uses a variable, i.e. SRH, that is not study specific and is commonly ascertained in aging studies, which facilitates not only the comparison of results between countries within our analyses but also the replicability of this study with findings from studies in different countries or in specific populations (eg, clinical samples); physical functioning measures that have been found to be directly comparable in these samples (29). Moreover, an integrative systematic data analysis approach was used (28). As Hofer and Piccinin (28) highlights another possible source of variation when performing replications or comparing results between studies is the different statistical procedures followed. In the present study, the exact same statistical procedure was followed in both samples reducing the possible variation due to methodological issues. Furthermore, a standardization procedure that not only has proved to be adequate to standardized health-related variables in aging studies but effectively equate the measures and allow the computation of the rates of change (22).

Some limitations should also be acknowledged. First, only two time points were used to compute the slopes because using all the time points between the two waves selected resulted in differential nonlinearity over time among studies and would have led to comparability issues. However, further research utilizing more than two time points of data within individuals in a single study would elucidate our understanding of the process of decline. Moreover, it would be interesting to develop further research that explores the potential heterogeneity in the patterns of change in these measures. Although in this study an overall pattern of decline was found, it is plausible that studies that focus on shorter follow-up periods and use more measurement occasions would identify improvers. The design and statistical procedure followed in the present study does not allow us to do this. Second, only individuals who were still alive at follow-up were considered in this study. Although this could be viewed as a limitation as it is plausible to expect that functional decline would be steeper in the years immediately prior to death (30), we would not expect this to cause differences in the ordering of the slopes, which is our main focus of interest. Third, although a wide range of measures were included in this study, only self-reported measures were included because they allow an integrated assessment of the individuals’ experience of loss and reflect the “lived experience.” It should be noted that research to date has highlighted their limitations compared to performance-based measures (31) as they may be affected by psychosocial (32) or cultural factors (25,29). However, for the purposes of this study that focuses on functional loss, these measures are appropriate although future research that takes account of differences in psychosocial and cultural factors, specifically personality traits or personal sense of control, may be interesting. Future studies should also consider including different grades of difficulty in ADLs and mobility measures. Finally, this study focused on samples from two Western countries, but further research is also needed to extend these comparisons to other populations from a diverse range of settings.

In summary, the results of the present study did not only provide further evidence supporting the hierarchy of loss in physical functioning found in previous research but also demonstrated that there is a hierarchy in the rates of decline in physical functioning over time and that this is consistently found in men and women from United States and England. Overall, our findings suggest that having difficulties when climbing several flights of stairs without resting, and stooping, kneeling, or crouching might be the first self-reported indicators of decline in physical functioning. These results were found in both American and English men and women. Prevention and intervention programs that aim to maintain physical functioning in older adults should develop activities that identify and address problems with climbing several flights of stairs, and stooping, kneeling, or crouching. The identification of some of these first indicators of decline in physical functioning could contribute to the design of new strategies to delay physical functioning decline in older adults.

## Supplementary Material

Supplementary data are available at *The Journals of Gerontology, Series A: Biological Sciences and Medical Sciences* online.

## Funding

Research reported in this publication was supported by the National Institute on Aging of the National Institutes of Health under award number P01AG043362 for the Integrative Analysis of Longitudinal Studies of Aging (IALSA) research network. The content is solely the responsibility of the authors and does not necessarily represent the official views of the National Institutes of Health. R.C. and E.G.W. are supported by the UK Medical Research Council (Programme code MC_UU_12019/4).

## Conflict of Interest

The authors declare that there are no conflicts of interest.

## Supplementary Material

Supplementary_figure_1_revised
