# Supplementary material for: Hierarchy and Speed of Loss in Physical Functioning: A Comparison Across Older U.S. and English Men and Women
Source: J Gerontol A Biol Sci Med Sci. 2016 Oct 17;72(8):1117–22. doi: 10.1093/gerona/glw209 (PMC5861940; doi:10.1093/gerona/glw209)
Supplement: Supplementary_figure_1_revised [file glw209_suppl_supplementary_figure_1_revised.docx]

HRS women ELSA women

HRS men ELSA men


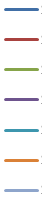
EAT

TOILET

DIME

CLIMB1

PUSH

CHAIR

STOOP

Supplementary Figure 1.

Rate of decline for men and women in both samples for selected measures.

Y-axis represent the scores of the standardized measures and in X-axis represents baseline and eight-year follow up.
